# Calcium-dependent adhesion protein CDH18, a potential biomarker for prognosis in uterine corpus endometrial carcinoma

**DOI:** 10.3389/fmolb.2025.1530253

**Published:** 2025-02-13

**Authors:** Xiaoxuan Tang, Shanxing Dang, Jie Qiu, Ruilan Zhou, Jing Ling, Limei Zhang, Xiaopeng Peng, Qingyun Li, Jin Liu, Wei Liao, Qingxiu Mei, Miao Xie, Yehong Sun, Jianmei Huang, Xuelian Du, Wencong Song

**Affiliations:** ^1^ The Fourth Clinical Medical College of Guangzhou University of Chinese Medicine, Shenzhen Traditional Chinese Medicine Hospital, Shenzhen, Guangdong, China; ^2^ School of Computer Science and Engineering, Yulin Normal University, Yulin, China

**Keywords:** uterine corpus endometrial carcinoma, CDH18, prognostic biomarkers, immune microenvironment, calcium-dependent adhesion protein

## Abstract

**Background:**

Uterine corpus endometrial carcinoma (UCEC) is one of the most common cancers in women, yet lacks specific and sensitive tumor markers for diagnosis, as traditional markers like CA125 show limited specificity. This study investigates the clinical significance and prognostic value of CDH18, a calcium-dependent adhesion protein linked to tumor progression, in UCEC.

**Methods:**

Clinical data from UCEC patients were sourced from The Cancer Genome Atlas (TCGA) database. Pan-cancer analysis, differential expression examination, and survival analysis were conducted to investigate the differential expression of the calcium associated protein-CDH18 and its prognostic relevance. CDH18 mutations in UCEC were examined using the cBioPortal database. Additional analyses included functional enrichment, tumor mutational burden, tumor microenvironment (TME) estimates via ESTIMATE, and immune infiltration assessment to clarify CDH18’s potential mechanisms in UCEC. Drug sensitivity testing was utilized to identify more suitable therapeutic options for patients. Immunofluorescence staining (IF) and Real-Time Polymerase Chain Reaction techniques (RT-PCR) confirmed CDH18 expression in UCEC tumor.

**Results:**

CDH18 expression was markedly increased in UCEC and showed a significant association with poorer prognosis, which was confirmed by our IF and RT-PCR results. Thirteen mutation sites were identified, and survival analysis showed that patients with higher CDH18 expression had shorter overall survival. The expression of CDH18 was confirmed to be an independent predictor of overall survival by multivariate COX regression analysis. Additionally, a predictive nomogram model was developed to accurately forecast outcomes for individuals with UCEC. Correlation analysis revealed that CDH18 expression exhibited a negative correlation with CD8 T cell levels and a positive correlation with resting NK cell and macrophage M2 levels. In the group with high CDH18 expression, the IC50 values for (5Z)-7-Oxozeaenol, AG-014699, CEP-701, Mitomycin C, PD-0325901, PD-0332991, PHA-665752, SL 0101-1, and SN-38 were notably elevated.

**Conclusion:**

CDH18 is a novel promising biomarker in UCEC, uniquely associating tumor progression, immune modulation, and chemotherapy resistance, offering enhanced prognostic accuracy and guiding individualized therapeutic strategies for improved patient outcomes.

## 1 Introduction

Uterine corpus endometrial carcinoma (UCEC) ranks as the fourth most common cancer among women (Siegel et al., 2023), with its overall incidence rising by 132% over the past 30 years due to factors such as increased obesity rates and an aging population (Oaknin et al., 2022). While many UCEC cases are identified at an early stage (Ferlay et al., 2013), the prognosis for high-risk endometrial cancers remains poor (Zheng et al., 2021), with 5-year overall survival (OS) rates between 20% and 25% for advanced stages (Creasman et al., 2006a). In terms of treatment, the standard surgical procedure involves total hysterectomy along with bilateral salpingo-oophorectomy (Wortman et al., 2019). Radiation and chemotherapy can be beneficial in specific treatment scenarios (Creutzberg et al., 2000; Nout et al., 2011). Furthermore, Grouping endometrial cancer by molecular characteristics helps predict patient outcomes but current methods are inconsistent and provide limited treatment guidance (Oaknin et al., 2022). Therefore, it is crucial to discover novel prognostic biomarkers that can accurately assess the risks and prognoses of individuals with UCEC and underlying therapeutic targets that can effectively guide personalized therapeutic approaches.

The CDH18 gene, which exhibits specific expression levels across various tumor types and the central nervous system, was reported to be related to tumor progression (Chmelarova et al., 2018). CDH18 expression is upregulated when constitutive photomorphogenesis 1 (COP1) is silenced, which promotes gastric cancer development by failing to inhibit PI3K/AKT signaling (Zhao et al., 2023). Additionally, CDH18 is predominantly expressed in the central nervous system, where it plays an essential role in calcium-dependent cell adhesion. Studies have shown that lower levels of CDH18 increase glioma cell invasion and migration, whereas higher expression reduces glioma cell resistance to chemotherapy (Bai et al., 2018). CDH18 also supports calcium-mediated intercellular adhesion (Nollet et al., 2000) and is actively engaged in biological processes like synaptic adhesion, axon growth, and pathfinding (Chen et al., 2017). Recent findings highlight an association between CDH18 and macrophages within the UCEC tumor microenvironment (Song et al., 2023).

Based on the observed association between CDH18 and immune cells such as macrophages in the tumor microenvironment, we hypothesize that CDH18 plays a critical role in immune modulation, potentially influencing tumor progression and differentiation through its impact on immune cell recruitment and polarization. Furthermore, given CDH18’s involvement in tumor progression and chemotherapy resistance in other cancer types, we propose that CDH18 expression levels could serve as a prognostic marker in UCEC. Considering the lack of consistent prognostic biomarkers in UCEC and the challenges associated with stratifying high-risk patients (Oaknin et al., 2022), CDH18 represents a promising candidate for personalized therapeutic approaches. Its potential role in both tumor progression and immune modulation warrants further investigation to establish its clinical relevance in UCEC.

## 2 Materials and methods

### 2.1 Collection of patient samples

Clinical data for endometrial cancer patients were obtained from The Cancer Genome Atlas (TCGA) database (https://cancergenome.nih.gov/), including 554 tumor samples and 35 normal tissue samples. The “limma” R software package was used to organize the data, and Patients without essential clinical details were excluded from the analyses (Ritchie et al., 2015; Liu et al., 2021). The information collected included patient gender, age, and tumor grade.

### 2.2 Pan-cancer analysis

CDH18 expression levels across various human cancers and corresponding normal tissues were compared using the TIMER database (https://cistrome.shinyapps.io/timer/). The gene expression distribution was visually represented using box plots to illustrate the results.

### 2.3 Differential expression analysis

The limma software package was used to assess differential gene expression between UCEC and normal samples in the gene expression matrix (Ritchie et al., 2015). Genes were categorized as differentially expressed (DEGs) if they met the criteria of |logFC| > 1 and a false discovery rate (FDR) < 0.05.

### 2.4 Survival analysis based on the expression of the calcium associated-protein CDH18 in UCEC

The TCGA-UCEC patients were stratified into two groups based on their CDH18 expression levels, namely the high expression group and the low expression group. Kaplan-Meier analysis was performed to evaluate survival differences by plotting survival curves with the “survival” and “survminer”s R packages, using the default median survival time and the “Surv_cutpoint” function (Li et al., 2020). The “survival” package was employed for univariate analysis to explore any associations between overall survival (OS) and CDH18 expression features. Multivariate analysis was subsequently conducted with the “survival” package to assess whether CDH18 expression could serve as an independent predictor of OS.

### 2.5 CDH18 mutations

The cBioPortal platform (http://cbioportal.org), which facilitates interactive exploration of cancer genomics data, was used to analyze mutations in CDH18 specifically in UCEC (Cerami et al., 2012).

### 2.6 Functional enrichment analysis

Differential expression analysis between the two groups was performed using DESeq2 in R, with DEGs defined by p < 0.05 and |log2(FC)| > 1. Spearman’s correlation analysis assessed the overlap between CDH18 expression and the top 11 DEGs. Gene Ontology (GO) and Kyoto Encyclopedia of Genes and Genomes (KEGG) pathway analyses were conducted on shared targets using the “clusterProfiler” R package with a p < 0.05 cutoff. Gene Set Enrichment Analysis (GSEA) was performed with the clusterProfiler package, and functions or pathways were deemed significantly enriched if the adjusted p-value was below 0.05 and the false discovery rate (FDR) was less than 0.25.

### 2.7 Tumor mutational burden (TMB), ESTIMATE of the tumor microenvironment (TME), and correlation with immune checkpoint genes

To explore the association between CDH18 expression, immune checkpoint (ICP) genes, and ESTIMATE scores in the TME, the SangerBox platform (http://sangerbox.com/Tool), which provides TCGA data analysis tools, was utilized (Wang D. et al., 2019). Yoshihara et al. created the algorithm known as ESTIMATE (Estimation of Stromal and Immune cells in Malignant Tumor tissues using Expression data) to predict tumor purity within the TME, incorporating stromal, immune, and overall estimate scores (Yoshihara et al., 2013). A p-value <0.05 was deemed statistically significant.

### 2.8 Proportions of immune infiltration cells in UCEC

The CIBERSORT deconvolution method was used to assess tumor-infiltrating immune cells (TIICs) in UCEC samples from the TCGA dataset (Chen et al., 2018). CIBERSORT generated a gene expression signature matrix for 22 types of TIICs, which was compared to the gene expression levels in TCGA samples. The resulting p-value indicated the reliability of the inferred proportions, and only samples with a CIBERSORT p < 0.05 were included in further analysis. The default number of permutations for the signature matrix was set to 100.

### 2.9 Drug sensitivity

The “pRRophetic” R package was utilized to predict the IC50 values of chemotherapeutic drugs. By evaluating the IC50 values across multiple targeted therapeutic drugs, a comprehensive understanding can be obtained regarding the differential responsiveness of distinct CDH18 expression cohorts to these drugs.

### 2.10 Cell lines and culture

Human endometrial cancer cells (HEC-1-B, Wuhan Pricella Biotechnology Co., Ltd., China) were used. These cells were cultured in a specific medium for HEC-1-B cells (GZ10605-500ML, Servicebio, China). Human endometrial epithelial cells (HEEC, FHHUM148, Fenghui Biotechnology, China) were cultured in DMEM/F12 medium supplemented with 10% fetal bovine serum (FBS).

### 2.11 Immunofluorescence staining

Immunofluorescence (IF) analysis of HEEC and HEC-1-B and Biotech (F116Ur01, Bioaitech, China) provided both paraffin sections of tissue samples from patients with UCEC and normal uterine endometrial tissue for immunofluorescence analysis. A brief experimental procedure can be found in our supplemental method file and the referenced studies (Zaqout et al., 2020; Song et al., 2015). The CDH18 antibody (No:13091-1-AP) was obtained from Proteintech.

### 2.12 Real-Time Polymerase Chain Reaction (RT-PCR)

Cellular samples were homogenized and lysed in the Trizol reagent. Total RNAs were isolated, and 2 μg RNA was reversely transcribed using High-Capacity cDNA Reverse Transcription Kit (Applied Biosystems, 4368813). Quantitative polymerase chain reaction was run on the QuantStudio™ 5 Real-Time PCR Instrument (Applied Biosystems) with β-actin (F- TGGCACCCAGCACAATGAA; R-CTAAGTCATAGTCCGCCTAGAAGCA) as an internal control. CDH18(F-TCCAAACTTCACTCTGAAGGACA; R-GGACAGGAAGGCTTCTGCAT) expression was analyzed by the comparative CT method. Please refer to online-only Data Supplement for the DNA sequences of primers.

## 3 Results

### 3.1 Differential expression of CDH18 in tumor and non-tumor tissues

**FIGURE 1:**
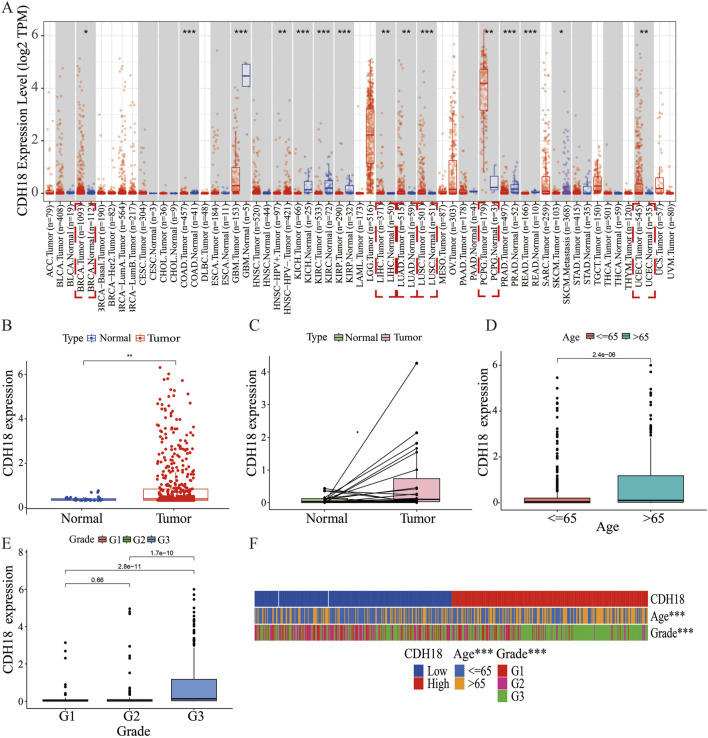
Expression of CDH18 in Various Tumors, Including UCEC. **(A)** CDH18 expression levels from the TCGA database for various cancer types. **(B, C)** Elevated CDH18 expression in UCEC tumor samples compared to non-tumor tissues. **(D, E, F)** Associations of CDH18 expression with age and grade in UCEC patients. (*P < 0.05, **P < 0.01, ***P < 0.001).

The expression of the calcium associated protein-CDH18 was assessed using data from the TCGA database for various cancer types. It was observed that CDH18 levels were elevated in numerous tumor tissues. Analysis from the TIMER database indicated that CDH18 expression was significantly higher in cancer types such as lung squamous cell carcinoma (LUSC), liver hepatocellular carcinoma (LIHC), lung adenocarcinoma (LUAD), pheochromocytoma and paraganglioma (PCPG), uterine corpus endometrial carcinoma (UCEC), and breast invasive carcinoma (BRCA) compared to adjacent normal tissues (Figure 1A). Specifically, in UCEC samples, CDH18 expression was notably higher than in non-tumor tissues (Figures 1B, C). Based on these findings, we hypothesize that CDH18 may be associated with tumor progression and potentially influence UCEC prognosis, although further studies are needed to confirm its functional role.

### 3.2 CDH18 as an independent prognostic biomarker for UCEC

**FIGURE 2:**
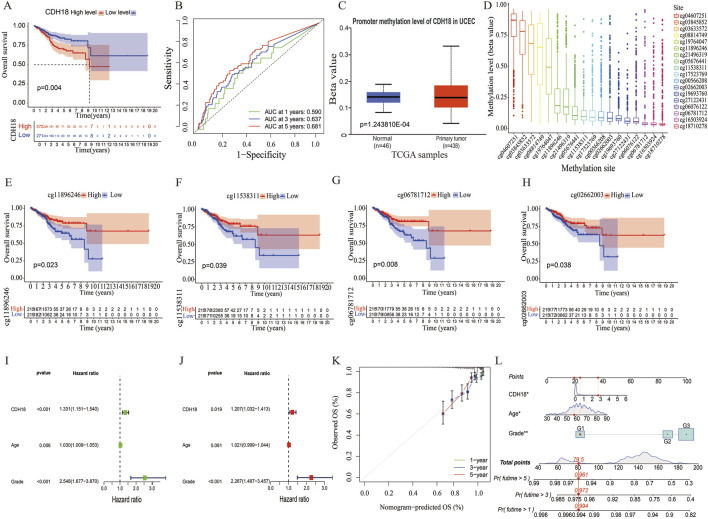
CDH18 as a Potential Independent Prognostic Biomarker for UCEC. **(A)** Kaplan-Meier survival curves for high versus low CDH18 expression groups. **(B)** ROC curves generated from TCGA data. **(C)** Comparison of CDH18 promoter methylation levels between tumor and normal tissues. **(D)** DNA methylation levels across various CpG sites. **(E–H)** Associations between UCEC prognosis and DNA methylation levels. **(I, J)** Univariate and multivariate Cox regression analysis of survival-related risk factors in UCEC. **(K, L)** Prognostic and calibration nomograms for predicting UCEC patient survival. (*P < 0.05, **P < 0.01, ***P < 0.001).

We also analyzed the association between the expression of the calcium associated protein-CDH18 and clinical factors such as age and histological grade in UCEC patients. Results indicated that patients aged over 65 exhibited significantly higher CDH18 levels compared to those under 65 (Figure 1D). Additionally, higher CDH18 expression was positively associated with advanced UCEC grades (Figure 1E). A heat map was generated to display the correlations among CDH18 expression, age, and grade in UCEC patients (Figure 1F). The prognostic significance of CDH18 was assessed further. As shown by Kaplan-Meier survival curves (p = 0.004) (Figure 2A), higher CDH18 expression was associated with poorer survival in UCEC. The area under the curve (AUC) values for 1-year, 3-year, and 5-year survival in the TCGA test cohort were 0.590, 0.637, and 0.681, respectively (Figure 2B). We also observed that CDH18 methylation status correlated with UCEC prognosis. CDH18 promoter methylation differed between tumor and normal samples from TCGA (Figure 2C), with 18 CpG sites showing increased DNA methylation (Figure 2D). Methylation levels at four CpG sites (cg11896246, cg11538311, cg06781712, and cg02662003) were significantly associated with survival outcomes (p < 0.05) (Figures 2E–H). The independence of clinical factors was evaluated through univariate and multivariate Cox regression analyses. Our analysis revealed that overall survival was significantly correlated with age (p = 0.006), grade (p < 0.001), and CDH18 expression (p < 0.001) (Figure 2I). Importantly, CDH18 expression emerged as an independent predictor for overall survival (Figures 2J, K). A calibration curve illustrating 1-, 3-, and 5-year survival nomograms demonstrated that the nomogram performed well, supporting CDH18’s utility in UCEC prognosis (Figure 2L).

### 3.3 CDH18 mutations in UCEC

**FIGURE 3:**
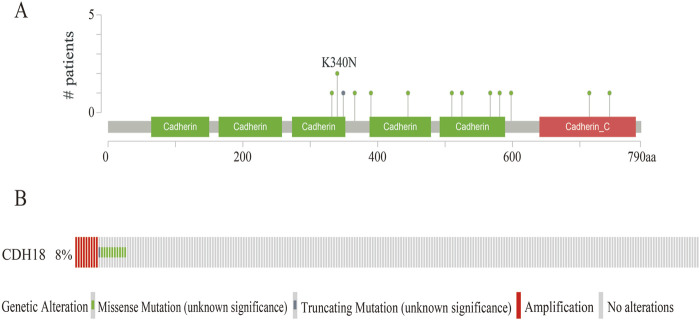
The Gene mutation of CDH18 in UCEC. **(A)**Mutation diagram of CDH18 in UCEC. **(B)** Diverse kinds of mutations in UCEC.

Through cBioPortal, 13 mutation sites located between amino acids 300 to 790 were detected, higher mutation accretion rate in K340N (Figure 3A). Besides, 8% CDH18 mutations occurred in UCEC, which included missense mutations, truncating mutations, and amplification (Figure 3B).

### 3.4 Co-expressed networks and potential function of CDH18 in UCEC

**FIGURE 4:**
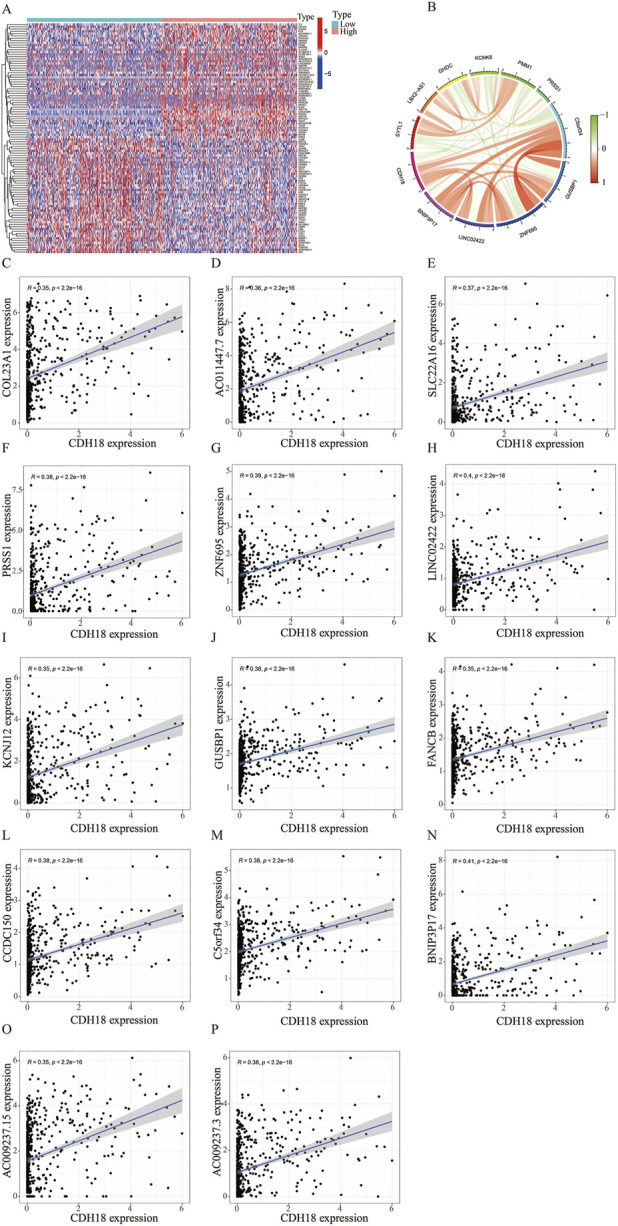
CDH18 Co-expressed Networks. **(A)** Heatmap showing differential expression of genes co-expressed (DEGs) with CDH18 in high and low expression groups (red: high; blue: low). **(B)** Circos plot visualizing co-expression networks of CDH18, with line colors representing correlation strength (R > 0.35, p < 2.2e-16). **(C–P)** Scatter plots illustrating significant positive correlations between CDH18 and key co-expressed genes.

**FIGURE 5:**
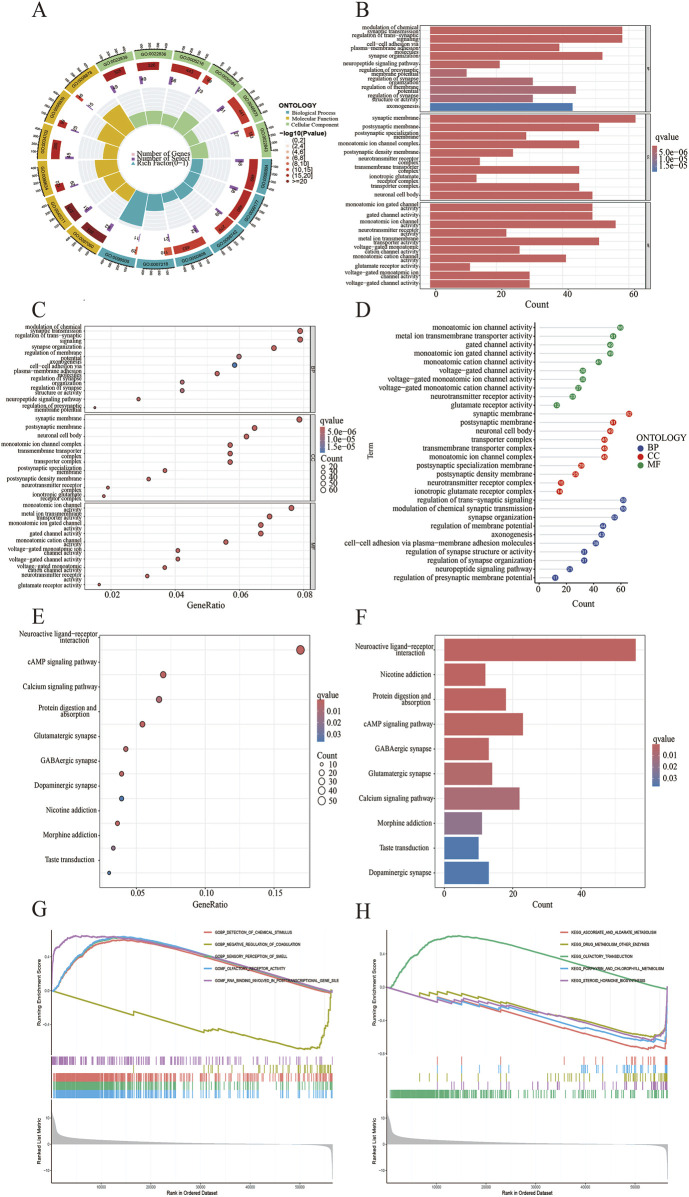
Functional Analysis of CDH18-associated DEGs. **(A)** GO-circle visualization for GO enrichment. **(B)** Barplot for GO enrichment. **(C, D)** Bubble plots showing GO enrichment results. **(E)** Bubble plot for KEGG pathways. **(F)** Barplot of KEGG pathways. **(G, H)** Gene Set Enrichment Analysis (GSEA).

To verify the potential function of CDH18 in UCEC, 1926 DEGs were screened, and the top 100 DEGs were present via heat map (Figure 4A). Moreover, the links between CDH18 and several important co-expressed genes were listed (Figure 4B). Examples of genes had positive correlation with CDH18 were displayed, such as BNIP3P17,LINC02422 and ZNF695 (R = 0.41,0.4,0.39 and P < 2.2e-16, 2.2e-16, 2.2e-16, respectively) (Figures 4C–P). GO analysis revealed that CDH18-related DEGs were primarily involved in chemical synaptic transmission modulation, trans-synaptic signaling regulation, synapse organization, and membrane-associated activities such as synaptic and postsynaptic membrane processes. Additionally, they were involved in neuronal cell body functions, monoatomic ion channel activity, metal ion transport across membranes, and gated ion channel activity (Figures 5A–D). KEGG pathway analysis further revealed that these DEGs were predominantly associated with neuroactive ligand-receptor interactions (Figures 5E, F). To investigate the hypothesis that elevated CDH18 expression may contribute to tumor growth, we conducted a GSEA. Results indicated that biological processes like detection of chemical stimuli, sensory perception of smell, and molecular functions such as olfactory receptor activity and RNA binding, particularly in posttranslational gene regulation, were significantly linked with increased CDH18 expression. In contrast, processes like negative regulation of coagulation were notably enriched in the low CDH18 expression group (Figure 5G). Genes linked to high CDH18 levels were active in sensory functions, while those linked to low levels were involved in breaking down drugs (Figure 5H).

### 3.5 CDH18 association with immune cell infiltration in TME of UCEC

**FIGURE 6:**
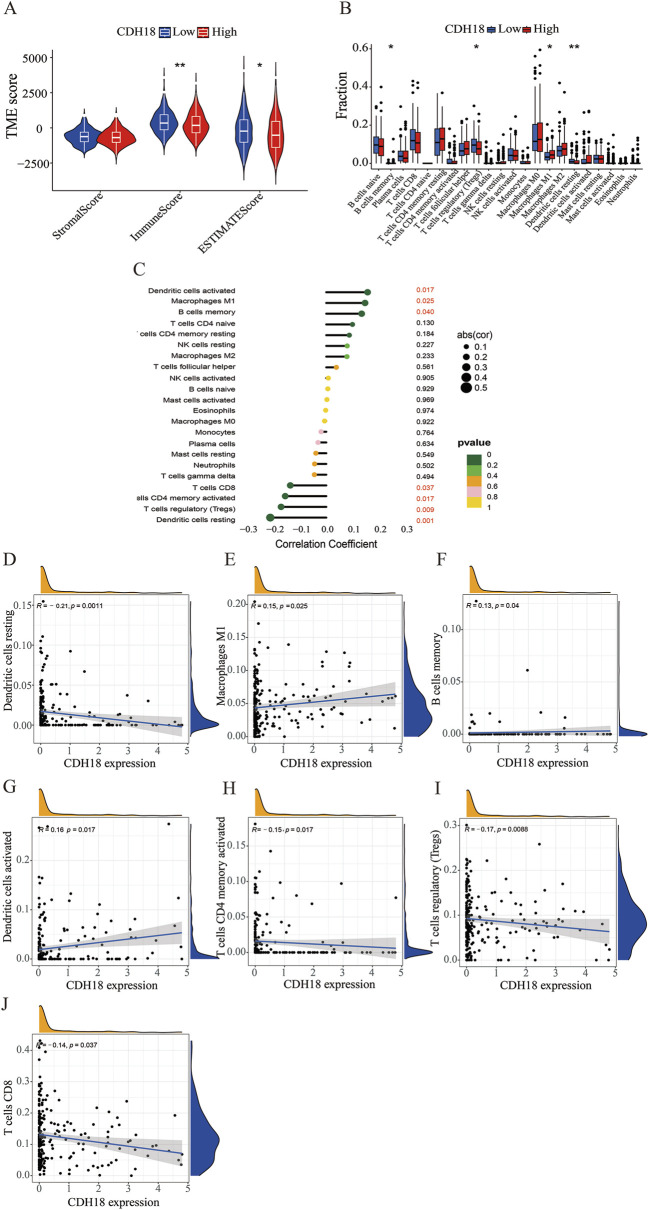
Tumor Microenvironment and Tumor-Infiltrating Immune Cell Distribution. **(A)** Box plot illustrating differences in the tumor microenvironment across CDH18 expression groups. **(B)** Violin plot displaying the distribution of various immune cell types. **(C–J)** Interaction between CDH18 and immune cell infiltration in UCEC.

Growing research indicates that components of the TME significantly influence the development and spread of tumors. We compared TME scores between high and low CDH18 expression groups, finding that the immune score was higher in the low CDH18 expression group, indicating that tumors with reduced CDH18 expression exhibit more immune cell infiltration and are associated with a better prognosis (Figure 6A). After confirming the link between CDH18 expression and the TME, we further examined the specific relationship between CDH18 and immune cell infiltration. As illustrated, CDH18 expression was altered across multiple immune cell types, including resting dendritic cells, M1 macrophages, regulatory T cells, and memory B cells (Figure 6B). Additionally, our findings suggest that patients with elevated CDH18 levels present an immunosuppressive microenvironment. CDH18 expression was positively correlated with resting NK cells and M2 macrophages, which promote tumor angiogenesis, growth, and metastasis (Danilo et al., 2016). Conversely, CDH18 expression showed a negative association with CD8^+^ T cells, known for inducing cell death through pathways like perforin-granzyme and Fas-Fas ligand (Wang W.1 et al., 2019) (Figures 6C–J).

### 3.6 CDH18 and immune checkpoints in UCEC

**FIGURE 7:**
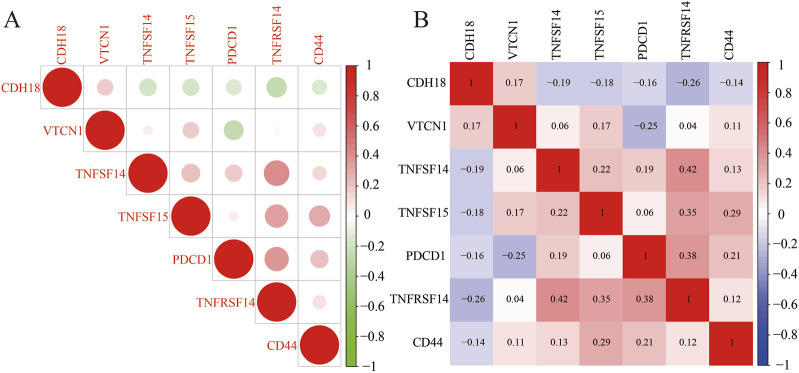
**(A, B)** Correlation analysis between CDH18 expression and immune checkpoint molecules in UCEC patients.

Under most circumstances, innate and adaptive immunity are generally effective in eliminating malignant cells. However, those cancer cells disguise themselves through immune checkpoint to evade the immune system. Of note, the CDH18 expression was proportional to VTCN1 (Sica et al., 2003; Cui and Li, 2016; Tringler et al., 2005; Tringler et al., 2006), while inversely proportional to TNFSF14, TNFSF15, TNFRSF14 (Dostert et al., 2019), PDCD1 (Okazaki and Honjo, 2007; Hassn Mesrati et al., 2021), and CD44 (Zhang et al., 2016), previous studies had showed that these genes mentioned above, including VTCN1 were immune checkpoint related-genes (Figures 7A, B). It was further revealed that CDH18 has a role in both the prognosis and therapeutic targets for UCEC treatment.

### 3.7 Relationship between CDH18 expression and drug sensitivity

**FIGURE 8:**
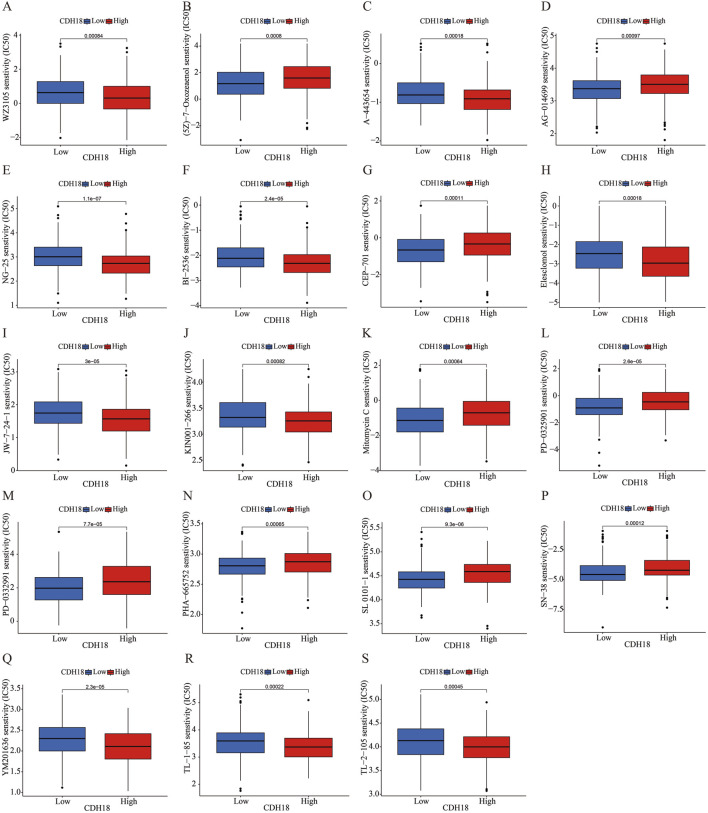
**(A–S)** Evaluation of IC50 values for various targeted therapies across CDH18 expression levels in distinct patient cohorts.

In order to identify many potentially effective drugs and small molecule compounds, we conducted an evaluation on the correlation between CDH18 expression and drug sensitivity. The IC50 values of (5Z)-7-Oxozeaenol, AG-014699, CEP-701, Mitomycin C, PD-0325901, PD-0332991, PHA-665752, SL 0101-1 and SN-38 were significantly elevated in the CDH18 high-expression group. These findings suggest that individuals with increased levels of CDH18 expression may exhibit limited response to therapeutic interventions provided by these medications (Figure 8). Collectively, the significant distinction observed among various groups with different CDH18 expression levels regarding IC50 value distributions for targeted drugs emphasizes the need for personalized medicine approaches.

### 3.8 Validation of CDH18 protein expression in UCEC samples

**FIGURE 9:**
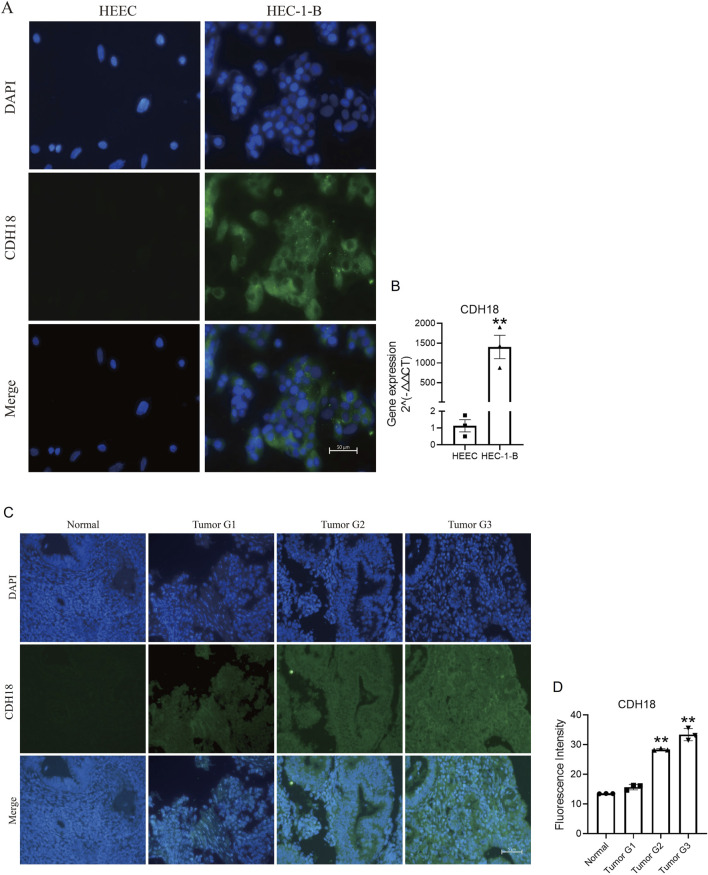
**(A)** Immunofluorescence images comparing CDH18 expression in endometrial cancer cells (HEC-1-B) with non-cancer cells (HEEC). **(B)** The gene of CDH18 expression in HEC-1-B and HEEC (RT-PCR data). **(C)** Immunofluorescence staining of CDH18 across UCEC grades I, II, and III in serial paraffin sections. **(D)** The statistics data of **Figure 9C.** The data ae presented as the mean ± SD, The p-values are calculated using two-tailed unpaired t-test, *p < 0.05, **p < 0.01.

To validate the findings, endometrial cells and paraffin tissue were subjected to immunofluorescence staining. The results confirmed a positive correlation of CDH18 expression in HEC-1-B endometrial cancer cell lines compared to HEEC of non-cancer cells (Figure 9A). Immunofluorescence analysis of paraffin tissue sections revealed progressively higher CDH18 expression levels with increasing WHO grades of UCEC, with staining predominantly localized to the cell membrane (Figure 9B).

## 4 Discussion

In this study, we explored the expression of Calcium-dependent adhesion protein CDH18 in UCEC and established its clinical relevance. We showed that high CDH18 levels are associated with advanced stages and poorer prognosis. These findings were further supported by immunofluorescence validation. Multivariate COX regression analysis reinforced CDH18 as an independent prognostic factor for overall survival in patients with UCEC, highlighting its potential as a novel biomarker for prognosis and a target for therapy.

The tumor microenvironment (TME) comprises tumor cells, stromal cells, endothelial cells, immune cells, and extracellular matrix components. These elements interact intricately with tumor cells, influencing their proliferation and progression (Xiao and Yu, 2020). To investigate underlying mechanisms, we observed that tumors with elevated CDH18 expression had reduced immune cell infiltration. CDH18 expression correlated positively with resting NK cells and M2 macrophages, both of which are known to inhibit anti-tumor immune responses (Zheng et al., 2021). Conversely, CDH18 expression was inversely linked to CD8^+^ T cells, which are capable of suppressing tumor growth. These findings underscore CDH18’s potential role in shaping the immune landscape within the TME, supporting our initial hypothesis.

A positive correlation was found between CDH18 and VTCN1, an immune checkpoint molecule associated with poor prognosis and cancer progression. In contrast, CDH18 showed negative correlations with TNFSF14, TNFSF15, PDCD1, TNFRSF14, and CD44, genes reported to play critical roles in tumor inhibition. Altogether, CDH18, as a calcium-related protein, appears to be closely linked to immune regulation in the tumor context.

Additionally, we assessed the relationship between CDH18 expression and anticancer treatment effectiveness, finding that CDH18 could potentially serve as a predictive marker for drug responsiveness. Specifically, IC50 values for drugs such as (5Z)-7-Oxozeaenol, AG-014699, CEP-701, Mitomycin C, PD-0325901, PD-0332991, PHA-665752, SL 0101-1, and SN-38 were notably higher in patients with elevated CDH18 levels, indicating that individuals with high CDH18 expression may exhibit reduced sensitivity to these agents. pRRophetic is an R-based bioinformatics tool designed to predict the sensitivity of cancer cells to various chemotherapeutic agents, typically quantified by the half-maximal inhibitory concentration (IC50) values (Geeleher et al., 2014). The package leverages publicly available gene expression and drug sensitivity datasets to construct predictive models, enabling the estimation of drug responses in new gene expression datasets (Gomis-Tena et al., 2020). IC50 (half-maximal inhibitory concentration) is a critical metric for assessing drug potency, representing the concentration required to inhibit a biological process (e.g., cell proliferation) by 50% under specific conditions (Zaqout et al., 2020). Lower IC50 values indicate higher drug efficacy against target cells. By predicting IC50 values, researchers can evaluate the potential sensitivity of different patient groups or cell populations to specific chemotherapeutic drugs, thereby informing personalized treatment strategies.

Interestingly, CDH18 appears to play varied roles in tumor progression across different cancer types. Our research found that high CDH18 expression is associated with poor prognosis in UCEC. KEGG pathway analysis indicated that CDH18-associated DEGs were predominantly linked to neuroactive ligand-receptor interactions, a pathway implicated in immunosuppression in colon cancer (Yang et al., 2023). This may partly explain why advanced stages and a poor prognosis in UCEC are associated with high CDH18 expression.

However, prior studies have reported conflicting roles for CDH18 in different cancers. For example, CDH18 was shown to inhibit invasion/migratory ability and chemoresistance via ubiquinol cytochrome c reductase core protein 2 (UQCRC2) in gliomas,and reduce malignancy in gastric cancer via the constitutive photomorphogenesis 1 (COP1)-PI3K/AKT pathway axis (Zhao et al., 2023; Bai et al., 2018). This variability suggests that CDH18 may engage different pathways in different cancer contexts. According to GO analysis across various tumor types, CDH18-associated proteins are mainly enriched in processes like modulation of chemical synaptic transmission, synaptic membrane activity, and monoatomic ion channel function. These functions may support tumor growth through synapse formation and contribute to malignancy by activating ion channels (Creasman et al., 2006a; Venkatesh et al., 2019; Venkataramani et al., 2019; Zeng et al., 2019).

Calcium-dependent adhesion proteins, particularly cadherins, play crucial roles in cancer progression and immune modulation. Role of Cadherins in Tumor Progression: cadherins, such as E-cadherin, are vital for maintaining normal cell adhesion. Loss of E-cadherin expression is associated with increased tumor cell motility and metastasis (Conacci-Sorrell et al., 2002). Cadherins interact with catenins to form adherents junctions, which are crucial for cellular signaling. Disruption of these pathways can lead to malignant transformation (Yu et al., 2019). Immune Modulation: Altered cadherin expression can affect immune cell interactions, facilitating tumor immune evasion. Tumor cells may exploit cadherin signaling to escape immune surveillance (Xu et al., 2018). Calcium Signaling: Calcium ions (Ca2+) are integral to immune responses and can modulate the activity of adhesion proteins, influencing both tumor progression and immune interactions (Alharbi et al., 2021).

Current studies on the direct association between CDH18 and immune cells or immune cell infiltration in cancer are relatively limited. Though our study provided a rudimentary knowledge of CDH18 in UCEC, the specific molecular mechanism of CDH18 in UCEC is still unclear. There are some limitations of using TCGA as a single data source for survival outcomes analysis. These limitations include data heterogeneity arising from variations in sample collection and processing methods, sample selection bias due to the non-random inclusion of cases, incomplete or outdated clinical annotations, and the lack of integration across multiple dimensions of data. TCGA provides a valuable resource for exploring genomic and clinical correlations, relying solely on it may compromise the generalizability and robustness of the findings. To address these challenges, we plan to incorporate data from additional databases and conduct experimental validations in future studies. Furthermore, we lack clarity on the molecular mechanisms of CDH18 in UCEC. Adding preliminary *in vitro* or *in vivo* functional studies to elucidate its role in tumor progression would strengthen the conclusions of our manuscript.

Therefore, both more clinical experiments and basic studies of CDH18 in UCEC are needed. In the next step, we aim to elucidate the specific molecular mechanisms of CDH18 in UCEC. By uncovering its functional role, CDH18 has the potential to serve not only as a biomarker for prognosis but also as a promising therapeutic target, particularly in the context of personalized medicine. Its modulation could pave the way for more tailored treatment strategies, including immunotherapy, thereby enhancing the precision and efficacy of clinical interventions for UCEC.

## Data Availability

The datasets presented in this study can be found in online repositories. The names of the repository/repositories and accession number(s) can be found in the article/supplementary material.
