# Supplementary material for: Calcium-dependent adhesion protein CDH18, a potential biomarker for prognosis in uterine corpus endometrial carcinoma
Source: Front Mol Biosci. 2025 Feb 13;12:1530253. doi: 10.3389/fmolb.2025.1530253 (PMC11864935; doi:10.3389/fmolb.2025.1530253)
Supplement: Supplementary file 1 [file DataSheet1.zip › suppmental file/supplemental methods.docx]

**Analysis Tools:**

The R packages ("**limma**" and "**survminer**") for differential expression and survival analysis.

To investigate the potential relationship between CDH18 expression levels and prognosis, we employed the "**limma**" R package for differential expression analysis and the "**survminer**" R package for survival analysis. The selection of these statistical tools was based on an extensive review of relevant literature, including studies such as PMID: 34605806, PMC7068734, PMC4402510. These studies have demonstrated the effectiveness and suitability of "limma" for robust differential expression analysis and "survminer" for detailed survival analysis, providing a solid scientific basis for their use in our study.

**Time-dependent ROC Curve:**

To comprehensively evaluate the contribution of CDH18 as a predictive marker for survival at different time points, we employed the "timeROC" and "survminer" R packages to generate time-dependent ROC curves. Specifically, the "timeROC" function was used to calculate the ROC curves at critical time points of 1 year, 3 years, and 5 years. These curves were then visualized using the "plot" function. This analytical approach allowed us to visually compare the predictive efficacy of CDH18 gene expression levels across different time points.

**Methylation Correlation Analysis:**

To investigate the correlation between CDH18 gene expression and methylation levels, we retrieved methylation data for the CDH18 gene from the TCGA database. For each methylation site, we used the "bioCor" function to calculate its correlation with CDH18 gene expression levels. The results of this correlation analysis were then meticulously visualized using the "ggplot2" and "ggpubr" R packages.

**Kaplan-Meier Curve (Figure 2A):**

The Kaplan-Meier curve in Figure 2A was generated using the default median survival time as the cutoff point, corresponding to a cumulative survival rate of 50%. Following the reviewer’s suggestion, we have provided the calculated cutoff point and included the risk table in the figure.

**IC50 Prediction and Drug Sensitivity Studies**

**pRRophetic** employs the following steps to predict IC50 values:

1. **Dataset Construction**: The package utilizes gene expression and corresponding drug sensitivity (IC50) data from cancer genome projects such as the Cancer Cell Line Encyclopedia (CCLE) [2] and the Genomics of Drug Sensitivity in Cancer (GDSC) [3]. These datasets form the training set for model development.
2. **Selection of Gene Expression Features**: During training, pRRophetic applies linear regression models, such as ridge regression, to identify gene expression features significantly correlated with drug sensitivity. These selected genes serve as input variables for the predictive model [4].
3. **Model Training and Validation**: Using the identified gene expression features, pRRophetic constructs predictive models and evaluates their performance through methods like cross-validation to ensure robustness and generalizability across different datasets [5].
4. **Prediction on New Data**: The trained models are applied to new gene expression datasets (e.g., patient samples) to compute predicted IC50 values for each sample against specific drugs [1].

**Data Sources and Methodology**

pRRophetic primarily relies on comprehensive cancer cell line databases such as CCLE [2] and GDSC [3], which provide extensive gene expression profiles and drug response data for numerous cell lines. Utilizing statistical learning techniques, including linear and ridge regression, pRRophetic establishes associations between gene expression patterns and drug sensitivities, facilitating accurate prediction of IC50 values in novel datasets [4].

**References**

[1] Geeleher, P., Cox, N., & Huang, R. S. (2014). pRRophetic: an R package for prediction of clinical chemotherapeutic response from tumor gene expression levels. *PLoS One*, 9(9), e107468. doi: <https://doi.org/10.1371/journal.pone.0107468>.

[2] Barretina, J., Caponigro, G., Stransky, N., et al. (2012). The Cancer Cell Line Encyclopedia enables predictive modelling of anticancer drug sensitivity. *Nature*, 483(7391), 603–607. doi: <https://doi.org/10.1038/nature11003>.

[3] Garnett, M. J., Edelman, E. J., Heidorn, S. J., et al. (2012). Systematic identification of genomic markers of drug sensitivity in cancer cells. *Nature*, 483(7391), 570–575. doi: <https://doi.org/10.1038/nature11005>.

[4] Ruppert, D. (2004). The Elements of Statistical Learning: Data Mining, Inference, and Prediction. *Journal of the American Statistical Association*, 99(466), 567. doi: <https://doi.org/10.1198/jasa.2004.s339>.

[5] Kuhn, M., & Johnson, K. (2013). Applied Predictive Modeling (2nd ed.). New York: Springer.

**Immunofluorescence Staining**

**For Paraffin Sections** [38]:

1. **Fixation**: Use paraformaldehyde to fix the tissue, preserving its structure and antigenicity.
2. **Deparaffinization and Rehydration**: For paraffin-embedded sections, remove the paraffin using organic solvents (xylene) and rehydrate the sections by immersing them in a series of graded ethanol solutions.
3. **Antigen Retrieval**: Restore the immunoreactivity of antigens through heat-induced epitope retrieval (HIER) or enzyme-induced epitope retrieval (EIER).
4. **Blocking**: Incubate the sections with a blocking solution (bovine serum albumin) to reduce non-specific background staining.
5. **Primary Antibody Incubation**: Incubate the sections with a specific primary antibody (CDH18, No:13091-1-AP，Proteintech) to bind the target antigen.
6. **Washing**: Wash the sections with a buffer solution (PBS) to remove unbound primary antibodies.
7. **Secondary Antibody Incubation**: Incubate the sections with a fluorophore-labeled secondary antibody that binds to the primary antibody（Alexa Fluor1 488 conjugate）, enabling fluorescence labeling of the target antigen.
8. **Second Washing**: Wash the sections with buffer to remove unbound secondary antibodies.
9. **Counterstaining**: Stain the cell nuclei with fluorescent dyes (Hoechst 33342) for localization under the microscope.
10. **Mounting**: Use an anti-fade mounting medium to cover the sections, protecting the sample and preserving fluorescence signals.
11. **Microscopy Observation**: Observe and capture images of the stained sections under a fluorescence microscope to analyze the expression and distribution of the target antigen.

**For culture cells** [39]:

Briefly, cells were cultured in 48-well plates and fixed with 4%formaldehyde for 10 min at room temperature. The cells were then washed with Phosphate Buffered Saline (PBS) for two times, and

5 min each. The cells were blocked with 1% BSA for 30 min at room temperature. Then, the cells were incubated with primary antibodies (CDH18, No:13091-1-AP，Proteintech) overnight at 4°C. The Secondary Antibody, Alexa Fluor1 488 conjugate was used according to the manufacturer’s instructions. Concurrently negative controls were stained with conjugated secondary antibodies alone. The cell nuclei were stained with Hoechst 33342. Observe and capture images of the stained sections under a fluorescence microscope to analyze the expression.

Quantitative analysis steps for Immunofluorescence Staining:

For tissue section immunofluorescence, use ImageJ software to measure the fluorescence intensity values.

**References**

[38]Zaqout, S., Becker, L. L., & Kaindl, A. M. (2020). Immunofluorescence staining of paraffin sections step by step. *Frontiers in Neuroanatomy*, 14, 582218. doi: <https://doi.org/10.3389/fnana.2020.582218>.

[39] Song, W., Mu, H., Wu, J., Liao, M., Zhu, H., Zheng, L., He, X., Niu, B., Zhai, Y., Bai, C., Lei, A., Li, G., & Hua, J. (2015). miR-544 regulates dairy goat male germline stem cell self-renewal via targeting PLZF. *Journal of Cellular Biochemistry*, 116(10), 2155–2165. doi: <https://doi.org/10.1002/jcb.25172>.
