# Supplementary figures and images for: Calcium-dependent adhesion protein CDH18, a potential biomarker for prognosis in uterine corpus endometrial carcinoma
Source: Front Mol Biosci. 2025 Feb 13;12:1530253. doi: 10.3389/fmolb.2025.1530253 (PMC11864935; doi:10.3389/fmolb.2025.1530253)

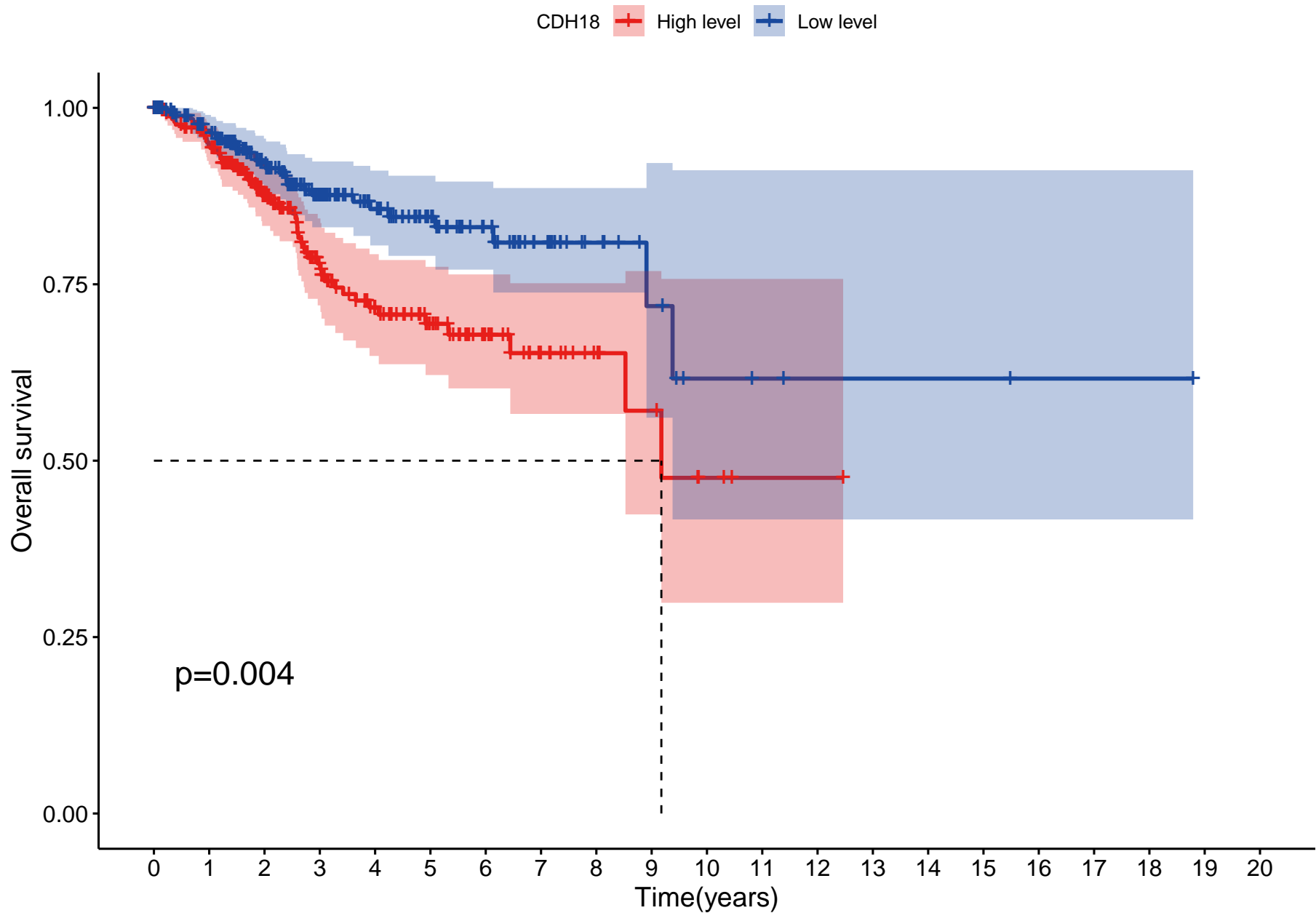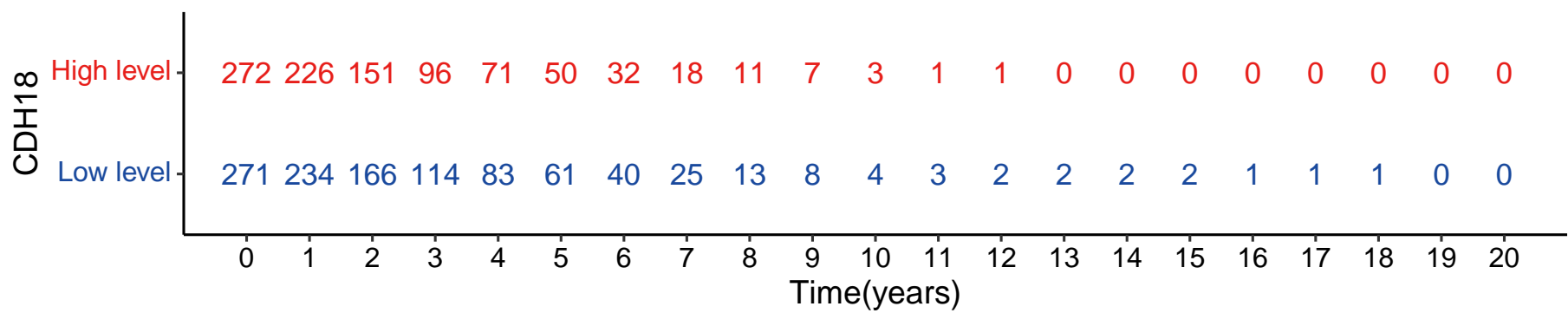

Supplement: Supplementary file 1 [file DataSheet1.zip › suppmental file/CDH18.surv.pdf]

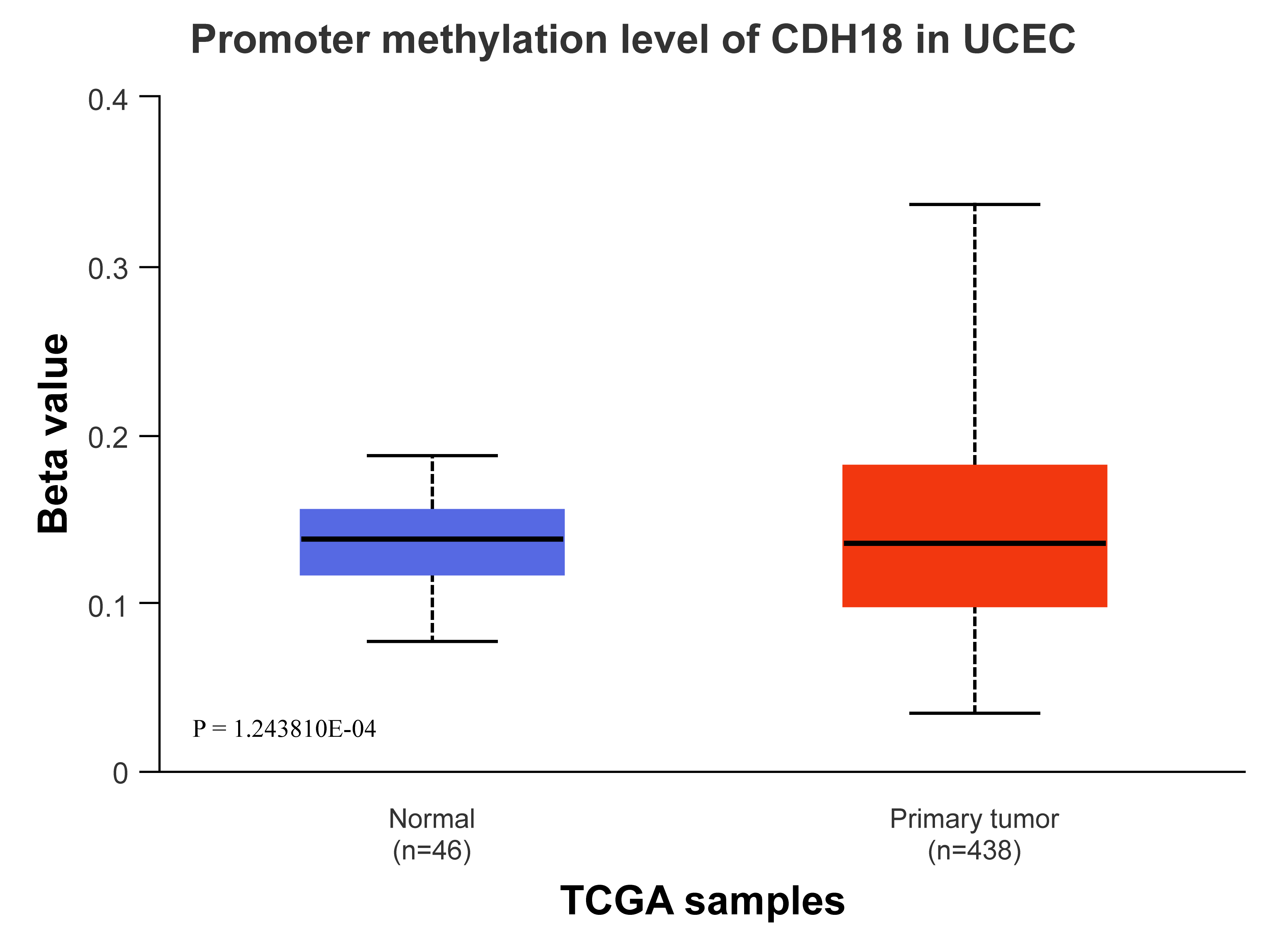

Supplement: Supplementary file 1 [file DataSheet1.zip › suppmental file/promoter methylatiion.tif]
